# The expression of retinal cell markers in human retinal pigment epithelial cells and their augmentation by the synthetic retinoid fenretinide

**Published:** 2011-06-25

**Authors:** Amanda-Jayne Carr, Anthony A. Vugler, Lu Yu, Maayan Semo, Pete Coffey, Stephen E. Moss, John Greenwood

**Affiliations:** 1 Department of Cell Biology, UCL Institute of Ophthalmology, University College London, London, UK; 2 Department of Ocular Biology and Therapeutics, UCL Institute of Ophthalmology, University College London, London, UK

## Abstract

**Purpose:**

In several species the retinal pigment epithelium (RPE) has the potential to transdifferentiate into retinal cells to regenerate functional retinal tissue after injury. However, this capacity for regeneration is lost in mammals. The synthetic retinoic acid derivative, fenretinide [*N(*4-hydroxyphenyl) retinamide], induces a neuronal-like phenotype in the human adult retinal pigment epithelial cell line (ARPE-19). These changes are characterized by the appearance of neural-like processes and the expression of neuronal markers not normally associated with RPE cells. Here we assess whether fenretinide can induce a neuroretinal cell phenotype in ARPE-19 cells, by examining retinal cell marker expression.

**Methods:**

ARPE-19 cells were treated daily with culture medium containing either 3 μM fenretinide or dimethyl sulfoxide as a control for 7 days. Cells were processed for immunocytochemistry, western blotting, and for analysis by PCR to examine the expression of a panel of RPE, neural, and retinal-associated cellular markers, including classical and non-canonical opsins.

**Results:**

Treatment with fenretinide for 7 days induced the formation of neuronal-like processes in ARPE-19 cells. Fenretinide induced the expression of the cone long wavelength sensitive opsin (OPN1lw) but not rhodopsin (RHO), while decreasing the expression of RPE cell markers. Many of the neuronal and retinal specific markers examined were expressed in both control and fenretinide treated cells, including those involved in photoreceptor cell development and the multipotency of neural retinal progenitor cells. Interestingly, ARPE-19 cells also expressed both photoreceptor specific and non-specific canonical opsins.

**Conclusions:**

The expression of retinal-associated markers and loss of RPE cell markers in control ARPE-19 cells suggests that these cells might have dedifferentiated from an RPE cell phenotype under standard culture conditions. The expression of molecules, such as the transcription factors paired box 6 gene (PAX6), sex determining region Y-box 2 (SOX2), cone-rod homeobox (CRX), and neural retina leucine zipper (NRL), further implies that in culture these cells are predisposed toward a retinal progenitor-like state. The fenretinide-induced increase in photoreceptor cell markers, accompanied by a decrease in RPE cell markers, suggests that retinoids may play a role in the transdifferentiation of RPE cells. Importantly, our data show for the first time the expression of a vertebrate ciliary opsin (OPN1lw) and rhabdomeric-like opsin, opsin 4 (OPN4 also known as melanopsin) in a clonal cell line. Together these data suggest that ARPE-19 cells are primed for and possess the capacity to differentiate toward a retinal cell-like lineage.

## Introduction

The retinal pigment epithelium (RPE), located between the neurosensory retina and the choroid, is a monolayer of cells vital for the maintenance of photoreceptor cell function and integrity. The RPE also plays a critical part in the development of the retina [1] and in some species participates in the regeneration of functional neural retinal tissue after injury [2] through RPE cell dedifferentiation, proliferation, and redifferentiation into the cellular elements of the retina (a process termed transdifferentiation). Many mature amphibians, such as the salamander [3], newt [4,5], and *Xenopus* [6], are capable of this process throughout their lives, while in birds it appears to be restricted to a limited period during embryonic eye development [7]. Mammals have lost the ability to regenerate the retina in vivo, although there is evidence to suggest that embryonic RPE cells transplanted into the adult eye are capable of forming retinal-like tissue [8] and that embryonic RPE cells are able to form a multilayered structure, expressing several retinal cell markers after treatment with basic fibroblast growth factor (bFGF) in culture [9].

To regenerate a complete retina, RPE cells must be able to transdifferentiate into several cell types. Analysis of cells from species where transdifferentiation has been shown to occur has revealed that RPE cells are capable of differentiating into retinal ganglion, amacrine, photoreceptor, and glial cells [5,9-11], and lens [4]. Many factors have been shown to regulate the transdifferentiation of RPE cells into neural retina, including bFGF [11], insulin [12], neuroD [13], mitogen activated protein kinase extracellular signal related kinase (MEK) [14], and neurogenin [15]. Whereas transforming growth factor (TGF)-β-like molecules, such as activin, thought to be involved in RPE cell differentiation [16], have been shown to block regeneration of the retina from RPE [17].

Retinoid signaling is crucial during the early stages of eye development [18,19] and is thought to promote the differentiation of photoreceptor cells [20]. Retinoic acid, a transcriptionally active vitamin A derivative, plays an integral role in the establishment of the retina and the specification of cells. Embryonic exposure to retinoic acid increases the incidence of rod photoreceptor cells at the expense of cone photoreceptors and amacrine cells in the zebrafish and rat retina, respectively [21,22], while morpholino-mediated knock-down of beta-carotene 15,15'-monooxygenase 1 (*bcox*), an enzyme critical to vitamin-A formation, in the embryonic zebrafish results in abnormal photoreceptor cell development [23].

It has previously been reported that retinoids have an effect on the differentiation of ARPE-19 cells, a spontaneously immortalized adult human RPE-derived cell line, which has been widely used as a model system for the study of RPE cells in vitro [24]. For example, treatment with retinoic acid delays the appearance of RPE cell morphology and the expression of RPE-specific genes peropsin, RPE65 and 11-cis retinal dehydrogenase [25]. Furthermore, there is compelling evidence to suggest that ARPE-19 cells are capable of differentiating into neuronal-like cells after treatment with the synthetic analog of retinoic acid *N*-(4-hydroxyphenyl) retinamide (fenretinide) [26] or after pretreatment with a neural stem cell media followed by 10 days exposure to retinoic acid [27]. Following retinoid treatment, cells display neuronal-like elongated morphology with the extension of multiple processes. Cells were characterized by immunocytochemistry and found to express several neuronal cell markers after fenretinide treatment, including neurofilament medium polypeptide (NF-M), neurofilament heavy polypeptide (NF-H), calbindin 2 (CALB2), neural cell adhesion molecule and microtubule associated protein 5 [26-28]. Furthermore, NSE and PAX-6 expression dimethyl sulfoxide (DMSO)-treated cells, suggesting that ARPE-19 cells may act as a neural progenitor cell line [28].

These findings suggest that a mechanism to activate transdifferentiation toward neural cells might exist, at least in part, in human RPE cells and raises the possibility that RPE could, with the appropriate manipulation, be induced to produce new retinal cell types, replacing those lost as a result of degenerative disease. Although Amemiya et al. were unable to induce the expression of the retinal-associated cell markers with retinoic acid using their protocol [27], the effects of the synthetic analog of retinoic acid, fenretinide, on RPE differentiation toward a neuroretinal cell fate have yet to be examined. In this study we have used reverse transcriptase PCR (RT–PCR), real-time quantitative PCR (Q-PCR), immunocytochemistry, and western blot analysis to examine the effects of fenretinide on the expression of retinal cell markers in ARPE-19 cells.

## Methods

### Cell culture

ARPE-19 cells (passage 22) and passage 2 human RPE cells (G.A. Limb, University College London, London, UK) were cultured in standard culture medium containing Dulbecco's Modified Eagle's Medium and Ham's F-12 Nutrient Mixture (DMEM/F12) mix (1:1)+ GlutaMAX™ with 1% penicillin–streptomycin and 10% fetal bovine serum (FBS; Invitrogen, Paisley, UK). The immortalized RPE cell line h1RPE7 was also cultured in Ham's F-10 with 10% heat-inactivated FCS, 2 mM l-glutamine and 1% penicillin-streptomycin [29]. All cells were incubated at 37 °C with 5% CO_2_. Cells were trypsinized and passaged onto 35-mm uncoated dishes and tissue culture flasks at a density of 2×10^3^ cells/cm^2^. The following day cells were washed with warm HEPES Balanced Salt Solution (Invitrogen), and the medium was replaced with low-serum medium containing DMEM/F-12 (1:1)+ GlutaMAX™ with 1% penicillin–streptomycin and 3% charcoal–dextran-treated FBS (Perbio Science UK Ltd., Thermo Fisher Scientific, Northumberland, UK). The following day cells were washed and treated with low-serum medium containing 3 μM fenretinide (Tocris Bioscience, Bristol, UK) dissolved in DMSO (Sigma-Aldrich Company Ltd, Dorset, UK). This treatment protocol was repeated once a day for a further 6 days. All handling of fenretinide was performed under low-light conditions. Control cells were cultured under identical conditions in low-serum medium with the addition of an equivalent volume of DMSO (0.03% volume [v]/v) only. Phase contrast images and scanning electron micrographs were taken following 7 days of treatment.

For the porcine lens capsule membrane (PLC) experiments the lens capsule was prepared as previously described [30]. Briefly, the anterior lens capsule was isolated from porcine eyes (Cheale Meats Ltd. Brentwood, UK). The polyester membrane was removed from a Corning® Transwell® insert (Sigma-Aldrich) and the anterior lens capsule glued to the remaining plastic support. The anterior lens capsule was carefully removed leaving the anterior lens capsule membrane attached to the plastic support. The insert was washed in Hanks Balanced Salt Solution containing 1% Penicillin-Streptomycin and tested for sterility before seeing of ARPE-19 cells. ARPE-19 cells were seeded onto tissue culture plastic or PLC and treated with DMSO or fenretinide, as described above.

### Reverse transcriptase PCR

Flasks of fenretinide-treated and control cells were twice washed in 1 x Dulbecco’s phosphate-buffered saline (2.7 mM KCl, 1.47 mM KH_2_PO_4_, 8.1 mM NaCl and 50.5 mM Na_2_HPO_4_ - PAA Laboratories Ltd., Yeovil, UK). TRIzol reagent (Invitrogen) was added to the flasks and cells lysed by trituration. RNA was isolated with chloroform induced phase separation and precipitated using isopropanol as instructed by the manufacturers. The RNA was treated with RQ1-RNase-free DNase (Promega, Southampton, UK) to remove any contaminating DNA. First-strand cDNA synthesis was performed on 3 μg of total RNA, using Superscript III Reverse Transcriptase with an oligo-(dT)20 primer (Invitrogen) at 50 °C, according to the manufacturer’s protocol. A reaction containing no reverse transcriptase was also prepared for each RNA sample as a control (-RT). Following cDNA synthesis all reactions were treated with RNase H (Invitrogen) to degrade the RNA template. PCR was performed on the first-strand cDNA synthesis reaction products, using GoTaq polymerase (Promega) according to the manufacturer’s protocol with gene-specific primers (synthesized by Eurofins MWG Operon, Ebersberg, Germany) described in Table 1. The PCR cycling parameters consisted of an initial denaturation step at 95 °C for 2 min, followed by 35 cycles of denaturation at 95 °C for 30 s, annealing at primer melting temperature minus 3 °C for 30 s and extension at 72 °C for 30 s, followed by a final extension step at 72 °C for 5 min. PCR products were separated on a 2% agarose gel and visualized using ethidium bromide. A no template water PCR control was included for each primer set.

**Table 1:** PCR primer sequences used for RT–PCR and Q-PCR.

**mRNA**	**Forward primer (5′-3′)**	**Reverse primer (5′-3′)**	**Accession number**	**Size**	**Tm-3 °C**
**RT–PCR**					
Best1	AGGTCGAATCCGGGACCCTA	GCACAACGAGGTCCAGCTCA	NM_004183.3	226	64
Brn3	CACCCTCCCTGAGCACAAGT	AGGCTAGGGGACAGCAAAGG	NM_006237	101	57
Calb2	GCGGCTACATTGACGAGCAT	GCAGCAGAAGCAGGGTTTGG	NM_001740	232	56
Crx	AGAGGGCAGGGAGCCAAATC	GCCAGTGTGTGGGGAAGAGG	NM_000554	236	58
Gfap	TCTGACCCTCTCCACCCCATA	GCCCTCCCAGTCCCATCTCT	S40719	288	58
Irbp	TGGAGCCCGACATCACTGT	TGCGGTCCTTGGCATTCTC	NM_002900	296	55
Krt8	AAGGATGCCAACGCCAAGTT	CCGCTGGTGGTCTTCGTATG	NM_002273	214	60
Nf-m	TGCTCCCTCCTCAGTCTTTGG	TCGTTTATTGTTTTTGGCTCAGTTG	BC096757	155	55
Nf-h	GCCAAGGTGGAGGTGAAGGA	TGGTCTGTGCTGGAGGATTTTT	NM_021076	271	55
Nse	GCTGCTCCTTGGCTTACCT	AACCCTGACGCTCCCATCAC	M22349	197	58
Nrl	AGAGCGCCTTCTGGTCCTAG	GCATCTCGGATAGAGGTCCT	NM_006177	420	60
Nr2e3	TGGTCCTCTTCAAGCCAGAGA	TTTCACCTCCACCCCCACTA	NM_014249	274	56
Opn1mw/lw	GCCCAGACGTGTTCAGCG	GACCATCACCACCACCAT	NM_000513	211	60
Opn1sw	GGTCACTGGCCTTCCTGG	TGCAGGCCCTCAGGGATG	NM_001708	174	60
Rho	TCAAGCCGGAGGTCAACAAC	TCTTGGACACGGTAGCAGAG	NM_000539	437	50
Opn3	CTCTTCGGGGTCACCTTTAC	AGGAGGAATCGTTGGCATC	NM_014322	330	56
Opn4	GAAGTCCTGAGCTCCCTGTG	TAACTGTTCCAGCGTGCAAA	NM_00100015	2009	52
Opn4 (2)	TGCGAGTTCTATGCCTTCTG	TGCGAGTTCTATGCCTTCTG	NM_00100015	462	53
Rcvrn	GGAAAAGCGAGCCGAGAAGA	CCTGGGGTGGATGTGTGTGT	NM_002903	282	56
Rgr	CAAGGGGGACAGAAACTTCACCAG	CTGCGGTGAGAGGCACTGCCAG	NM_002921	338	60
Rlbp	GCTGCTGGAGAATGAGGAAACTC	GGCTGGTGGATGAAGTGGAT	NM_000326	173	52
Rrh	TCAGCTCGGTGGTGTCTTTG	GTCTCGGATTTCCCAAGCAA	NM_000280	286	54
Rpe65	GCCCAGGAGCAGGACAAAAG	GCGCATCTGCAAGTTAAAACCA	NM_000329	246	52
Sag	CTGCACCTTGCGGTCTCTCT	TTTTTCTTGCGCTTCCTCCA	NM_000541	195	52
Silve	GTGGTCAGCACCCAGCTTAT	GAGGAGGGGGCTATTCTCAC	NM_006928	233	52
Syp	CAGGGTGGGGCTTAGAATGG	GTGTGTGTGGTGGGGTGCTT	NM_003179	264	58
Tbp	GAACCACGGCACTGATTTTC	CCCCACCATATTCTGAATCT	NM_003194	157	52
Thy-1	GACCCACAGCCTCCAAGTCA	CCCGCAGAAGTCCCTGAGAA	NM_006288	178	58
Tubb3	GCGAGATGTACGAAGACGAC	TTTAGACACTGCTGGCTTCG	NM_006086	420	60
Tyr	TGCCAACGATCCTATCTTCC	GACACAGCAAGCTCACAAGC	NM_000372	316	52
**Q-PCR**					
Crx	CCACTATTCTGTCAACGC	CCAAACCTGAACCCTGG	NM_000554	232	60
Krt8	AAGGATGCCAACGCCAAGTT	CCGCTGGTGGTCTTCGTATG	NM_002273	214	60
Opn1mw/lw	GCCCAGACGTGTTCAGCG	GACCATCACCACCACCAT	NM_000513	211	60
Opn3	CTCTTCGGGGTCACCTTTAC	AGGAGGAATCGTTGGCATC	NM_014322	330	56
B2m	TGTTGATGTATCTGAGCAGGTTG	AAGATGTTGATGTTGGATAAGAGAATC	NM_004048	100	60
Gapdh	CCCCACCACACTGAATCTCC	GGTACTTTATTGATGGTACATGACAAG	NM_002046	104	60
Tubb	AATCCCCACCTTTTCTTACTCC	AAAGATGGAGGAGGGTTCCC	NM_178014	119	60

Primer sequences for amplification of human opsins, RPE, retinal and neuronal specific genes: class IV POU domain-containing transcription factor 1 (Brn3), Calbindin2 (Calb2), Cone rod homeobox (Crx), Glial fibrillary acidic protein (Gfap), Interphotoreceptor retinol binding protein (Irbp), Neurofilment medium and heavy chains (Nf-m and Nf-h), Neuron specific enolase (Nse), Neural retinal leucine zipper (Nrl), retinal specific nuclear receptor (Nr2e3), Medium and long wave cone opsin (Opn1mw/lw – primer pair amplifies both medium and long wavelength opsin due to highly conserved sequence), short wave cone opsin (Opn1sw), Rhodopsin (Rho), Encephalopsin (Opn3), Melanopsin (Opn4 – amplified in cells using nested PCR), Recoverin (Rcvrn), Retinal G protein coupled receptor (Rgr), retina arrestin (Sag), Synaptophysin (Syp), Thy-1 cell surface antigen (Thy-1), b-3-tubulin (Tubb3), cytokeratin8 (Krt8), b-2-microglobulin (B2m) and Glyceraldehyde-3- phosphate dehydrogenase (Gapdh). The primer sequences used to amplify Crx for Q-PCR were taken from Peng and Chen 2007 [73].

### Real-time quantitative PCR

cDNA was prepared from ARPE-19 cells as described above, and quantitative PCR performed on a 7900HT Fast Real-Time PCR System (Applied Biosystems, Warrington, Cheshire, UK). Triplicate PCR reactions were prepared using 1 μl cDNA with Power SYBR Green PCR master mix (Applied Biosystems) and 0.2 μM of gene-specific primer (Eurofins MWG Operon) in a total volume of 25 μl. Primer sequences are contained in Table 1. Data were analyzed using SDS 2.2.2 (Applied Biosystems), and raw fluorescence data were exported into the DART–PCR spreadsheet (version 1.0) [31] to calculate relative gene expression normalized to the geometric mean of glyceraldehyde-3-phosphate dehydrogenase (*GAPDH*), β-tubulin, and β-2-microglobulin. The specificity of all primers was assessed by gel electrophoresis of amplified products, band sequencing, and examination of the dissociation curve. The effects of fenretinide treatment on relative mRNA expression levels were assessed using SigmaStat 3.5 software (Systat Software, Inc., Chicago, IL). Two-tailed Student *t* tests were performed to compare expression in DMSO- and fenretinide-treated cells (n=4 per treatment group).

### Immunocytochemistry

After 7 days of fenretinide or DMSO treatment, cells were washed with 0.1 M PBS (138 mM NaCl, 3.89 mM KCl, 2.13 mM KH_2_PO_4_, 8.16 mM Na_2_HPO_4_) fixed in 4% paraformaldehyde in PBS for 30 min at 4 °C and blocked for 2 h at 4 °C in a PBS solution containing 0.3% Triton X-100 (PBS-TX) and 5% normal donkey serum (NDS; Stratech Scientific Ltd., Newmarket, UK). Cells were then incubated overnight at 4 °C in PBS-TX containing 1% NDS with primary antibodies raised in mouse: RHO clone 4D2 (1:100, R Molday, University of British Columbia, Vancouver, Canada), KRT8 (1:2,000; Millipore, Watford, UK), CRX (1:1,000; Abnova, Heildelberg, Germany), NSE (1:50; Cymbus Biotechnology, Hampshire, UK), SYP (1:5,000; Millipore (UK) Ltd., Watford, UK), NF-M (1:1,000; Millipore UK) and rabbit: NF-H (1:5,000; Millipore UK), SCN1a (1:1,000; Millipore UK), PAX6 (1:300; Covance, Princeton, NJ), OPN1mw/lw (polyclonal antisera JH492; J. Nathans, John Hopkins University, Baltimore, MD), CALB2 (1:1,000; Swant, Bellinoza, Switzerland), RCVRN (1:1,000; Millipore UK), THY-1 (1:500; Source Bioscience AUTOGEN, Nottingham, UK), and OPN4 (antiserum, 1:10,000 and blocking peptide N-terminal [15AA NH_2_-MNPPSGPRVPPSPTQ-COOH diluted at 100 ng/ml and pre-absorbed overnight at 4 °C before application] I. Provencio, University of Virginia, Charlottesville, VA). The following day cells were washed in PBS before incubation with appropriate combinations of FITC- or TRITC-conjugated antibodies (Stratech Scientific Ltd.) diluted at 1:200 in PBS-TX with 2% NDS. Cells were counterstained with 4’6-diamindino-2-phenylindole dihydrochloride (Sigma-Aldrich), washed in PBS and mounted in Vectorshield (Vector Laboratories Ltd., Peterborough, UK). Staining was imaged and analyzed using a Zeiss 510 confocal microscope with LSM Image Browser software (Joel (UK) Ltd., Welwyn Garden City, UK). As a control for the specificity of secondary antibodies, primary antibodies were omitted in some dishes.

### Western blot analysis

Flasks of fenretinide- and control DMSO-treated cells were placed on ice, washed twice in cold 1X Dulbecco’s phosphate-buffered saline and harvested by scraping in lysis buffer (10 mM HEPES, 1% Triton X-100, 150 mM KCl, 1 mM PMSF, 10 ng/ml leupeptin, 1 mM dithiothereitol (DTT), 50 ng/ml aprotonin, 10 mM NaF, 100 μM sodium vanidate). The solutions were mixed at 4 °C for 30 min on a tube rotator and centrifuged at 17,000 xg for 30 min. The aqueous supernatants were isolated and the protein concentration estimated using BioRad protein assay reagent (Biorad, Hemel Hempstead, UK). Samples were diluted 1:1 in Laemmli sample buffer and denatured at 95 °C for 5 min. Equal amounts of protein were separated by sodium dodecyl sulfate PAGE (SDS–PAGE) and transferred to Hybond-polyvinylidene fluoride (PVDF) membrane (GE Healthcare Life Sciences, Buckinghamshire, UK) by electrophoresis at 4 °C. Membranes were blocked at room temperature for 1 h in blocking solution containing 10% milk in TBS-0.1% Tween-20 (TBS-T). Membranes were incubated overnight at 4 °C in 10% milk/TBS-T containing primary antibodies raised in mouse: CRX (1:1,000; Abnova) and KRT8 (1:2,000; Millipore UK); rabbit: CALB2 (1:10,000; Swant), OPN3 (1:500; Abcam, Cambridge, UK), PAX-6 (1:100; Source Bioscience AUTOGEN, Millipore UK), TUBB3 (1:2,000; Millipore UK), and OPN4 (1:10,000, I. Provencio); and goat: SOX2 (1:500; Source Bioscience AUTOGEN). The following day membranes were washed and incubated with horseradish peroxidase-conjugated secondary antibodies (1:2,000; Dako UK Ltd., Cambridgeshire, UK) in 1% milk/TBS-T for 2 h at room temperature. Protein signal was detected by incubation in LumiLight western blotting solution (Roche Products Ltd., Welwyn Garden City, UK) followed by exposure to autoradiographic film. Membranes were stripped using 8 M guanidium-HCl and reprobed with anti-GAPDH (1:1,000, goat polyclonal; Everest Biotech Ltd., Oxfordshire, UK). Protein levels were quantified by densitometry using the ImageJ processing program [32] and normalized to the GAPDH control for each membrane. Protein levels were analyzed using SigmaStat 3.5 software. Two-tailed Student *t* tests were used to compare protein levels in DMSO- and fenretinide-treated cells (n=4 per treatment group).

## Results

### Morphological changes induced by fenretinide

**Figure 1:**
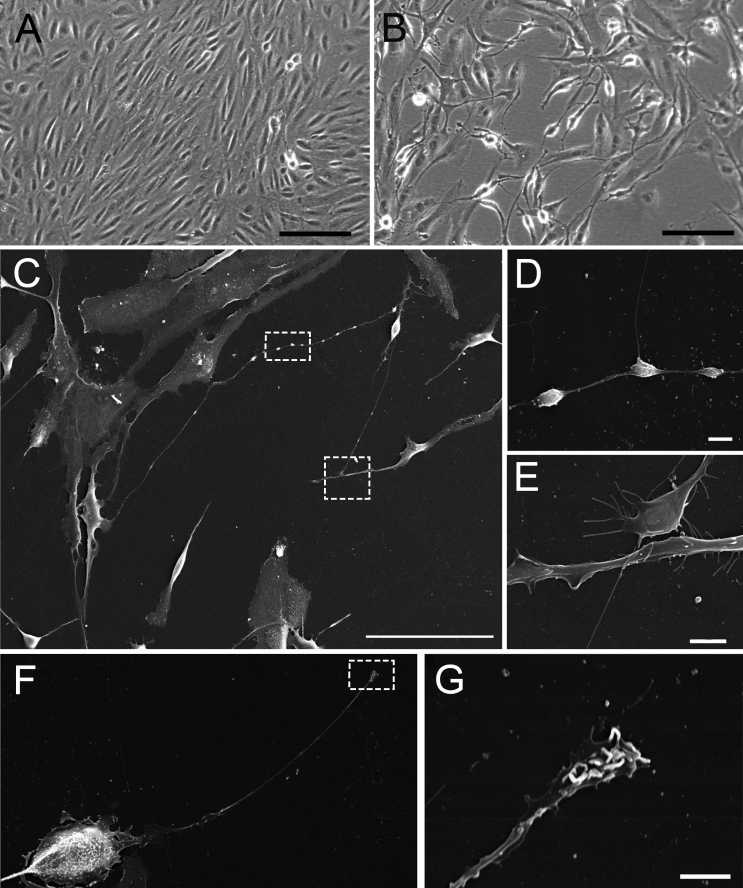
Fenretinide treatment induces a neuronal-like phenotype in ARPE-19 cells. Cells were treated on uncoated tissue culture plastic with media containing dimethyl sulfoxide (**A**) or 3 μM fenretinide (FR**; B**) once a day for 7 days, and the morphology was examined by phase microscopy. **C**: Scanning electron microscopy of FR-treated cells shows neural processes and the formation of varicosities and neurites (highlighted in the dashed boxes and shown at high magnification in **D** and **E**, respectively). **F:** Synaptic-like appositions were also present at the neurite terminal, shown at high magnification in **G**. Scale bars equal 200 μm in **A** and **B**, 100 μm in **C**, 4 μm in **D**, 20 μm in **F**, and 2 μm in **E** and **G**.

Initially we examined morphological changes associated with fenretinide treatment to ensure that the protocol used induced neuronal-like differentiation in the ARPE-19 cells. Similar to previous findings [26], cells cultured with DMSO appeared normal and grew to confluence (Figure 1A). In contrast, cells treated with 3 μM fenretinide for 7 days exhibited dramatic morphological changes characterized by an elongated appearance and the emergence of neuron-like processes (Figure 1B). Using scanning electron microscopy to resolve the morphology further, we observed structures reminiscent of neuronal cultures, including the formation of varicosities along the processes, the appearance of neurite-like branching/synaptic-like appositions (Figure 1C-E), and the presence of lamellae or ruffles at the “neurite” terminal (Figure 1F-G).

### Analysis of gene expression in fenretinide-treated cells

**Figure 2:**
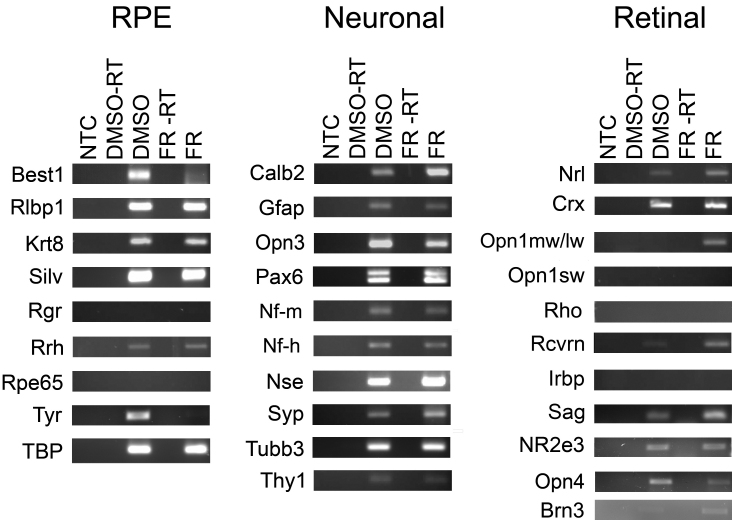
Analysis of cell markers in ARPE-19 cells by reverse transciptase PCR. Dimethyl sulfoxide (DMSO)- and fenretinide (FR)-treated cells express mRNAs normally associated with neuronal and retinal cells in culture and lose retinal pigment epithelium (RPE) cell markers. mRNA expression was detected in DMSO- and FR-treated cells using reverse transcriptase PCR. Primer pairs based on human opsins, and retinal, neuronal, and RPE cell marker sequences were used to amplify products from equal amounts of cDNA. A no-template RNA control (NTC) and a reaction lacking reverse transcriptase (-RT) was included for each amplification. *Tbp* was amplified as a positive loading control.

To test for the presence of mRNA expression induced by fenretinide, we isolated RNA from fenretinide- or DMSO-treated ARPE-19 cells, synthesized cDNA, and used a panel of primers to amplify several photoreceptor-, neuroretinal-, neuronal-, and RPE-specific cell markers in equal amounts of cDNA by RT–PCR (Figure 2). Amplification products were observed in both DMSO- and fenretinide-treated cells for photoreceptor cell markers *Rcrvn*, *Sag,* and *NR2e3* and neuroretinal cell markers *Thy-1*, *Opn4*, *Calb2,* and *Gfap*. *Opn3* (also known as encephalopsin/panopsin), a non-canonical opsin expressed in the retina and other tissues throughout the body, was also present in both treatment groups. The neuronal cell markers *Nf-m*, *Nf-h*, *Nse*, *Syp,* and *Tubb3* and the RPE cell markers *Rlbp1*, *Krt8*, *Silv* (also known as *Pmel17*), and *Rrh* were expressed in cells with and without fenretinide treatment, along with genes involved in retinal cell differentiation, *Pax6*, *Crx,* and *Nrl*. Interestingly, primers used to amplify both cone opsin sequences (*Opn1mw* and *Opn1lw*) produced a product only in the fenretinide-treated cells; sequencing of the amplicon revealed it to be *Opn1lw*. The retinal ganglion cell marker *Brn3* was also amplified in fenretinide-treated cells only. Transcripts for the RPE cell markers *Best1* and *Tyr* were not detected following fenretinide treatment, and we were unable to detect *Rho*, *Opn1sw*, *Irbp*, *Rgr*, or *Rpe65* gene expression in either treatment group by RT–PCR.

**Figure 3:**
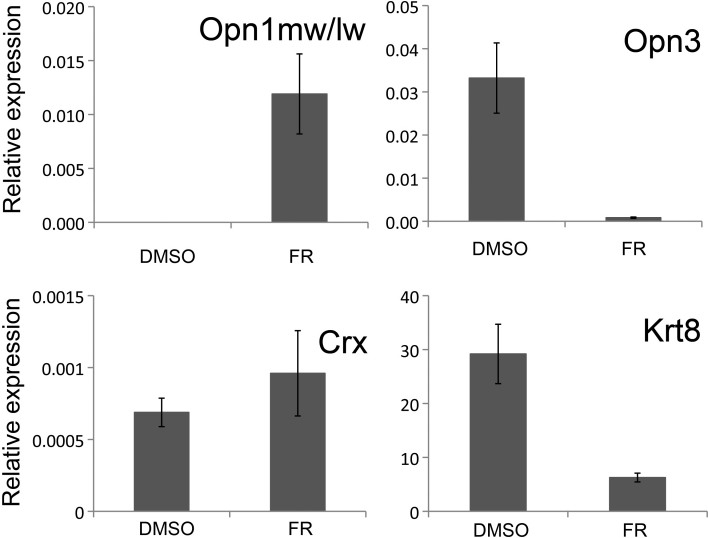
Quantitative analysis of gene expression in human retinal pigment epithelial cell line (ARPE-19) cells after fenretinide treatment. Real-time PCR was employed to examine quantitative differences in mRNA expression between dimethyl sulfoxide (DMSO)- and fenretinide (FR)-treated cells. Data shown are mean relative expression levels±standard error of the mean after normalization to the geometric mean of *Gapdh*, *Tubb,* and *β2m* (n=4).

To address quantitative changes in gene expression, we used Q-PCR to evaluate a select range of markers identified in the initial PCR screen (Figure 3). There was a significant increase in the expression of the *Opn1mw/lw* transcript (p<0.05) after fenretinide treatment, with complete absence of amplification in the DMSO-treated cells. *Opn3* and *Krt8* were expressed in both treatment groups; however there was a significant decrease in mRNA expression of both genes following fenretinide treatment (p<0.05). *Crx* was amplified in both treatment groups with no significant difference in the levels of mRNA expression after fenretinide treatment (p=0.42). *Rho* expression was not quantifiable in either treatment group (data not shown).

### Effect of fenretinide on protein expression in human retinal pigment epithelial cells

**Figure 4:**
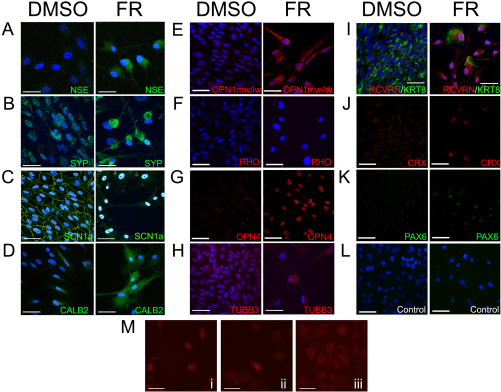
Immunocytochemical analysis of neuronal and photoreceptor cell markers in ARPE-19 cells after treatment with dimethyl sulfoxide or fenretinide. **A-K**: ARPE-19 cells were treated with 3 μM fenretinide or DMSO and processed for immunocytochemistry. The nuclei of cells are stained with 4',6-diamidino-2-phenylindole (blue) with the exception of cells where protein expression was detected within the nucleus (OPN4, CRX, and PAX6). Staining is indicated by the text color. **L**: Staining was not detected in control plates where only secondary antibodies were used. **M**: Final cell density did not affect cell morphology or changes in protein expression. ARPE-19 cells were seeded at various densities (i, 0.5×10^3^; ii, 1×10^3^; and iii, 2×10^3^ cells/dish) and cultured for 7 days in DMSO-containing medium before staining for CALB2 (red). All scale bars are 50 μm.

We next investigated the effects of 7 days treatment with DMSO or fenretinide in ARPE-19 cells on protein expression by immunocytochemistry. Several neuronal cell markers were expressed in ARPE-19 cells. The expression of NSE was enhanced by fenretinide treatment, particularly within the perinuclear region (Figure 4A). Both control and fenretinide-treated cells expressed SYP, with a wide distribution of staining throughout the control cells and clear staining throughout the dendrites after fenretinide treatment (Figure 4B). The voltage-gated Na^+^ channel SCN1a, which functions in the initiation and propagation of action potentials, was junctional in DMSO-treated cells. This pattern of expression was not maintained after fenretinide treatment; protein was localized to the nucleus, and sparse staining was seen along cytoplasmic extensions (Figure 4C). CALB2 staining was observed throughout the cytoplasm and along dendrites after fenretinide treatment; in the control dishes staining was less intense and perinuclear (Figure 4D). We then examined retinal cell- and retinal progenitor cell-associated markers. The expression of OPN1mw/lw was increased after fenretinide treatment and was observed throughout the cell (Figure 4E). Rhodopsin protein was not observed in ARPE-19 under either condition (Figure 4F). The OPN4 antibody detected a nuclear signal in both treatment groups with punctate nuclear expression in control cells, which was more intense after fenretinide treatment (Figure 4G). Pretreatment with the OPN4 blocking peptide blocked the signal (data not shown). TUBB3, a retinal cell-lineage marker found in early phase retinal cells (cones, horizontal, amacrine, and retinal ganglion cells), was sparsely distributed throughout the DMSO-treated cell cytoplasm, the staining intensity was increased after fenretinide treatment (Figure 4H). RCVRN was observed in a few control cells but was increased after fenretinide treatment; this was concurrent with a decrease in KRT8 expression (Figure 4I). PAX6 and CRX staining was detectable in the nucleus under both conditions but appeared more intense after fenretinide treatment (Figure 4J-K). Negative control dishes, where the primary antibody was omitted, produced no signal (Figure 4L). One consequence of treatment with fenretinide was a lower final density of cells in the dish at 7 days. By plating ARPE-19 cells at various densities and incubating in DMSO containing low-serum media only, we were able to demonstrate that the changes in ARPE-19 cell morphology and protein expression (CALB2) observed following fenretinide treatment were due to biochemical induction by fenretinide rather than differences in final cell density (Figure 4M).

**Figure 5:**
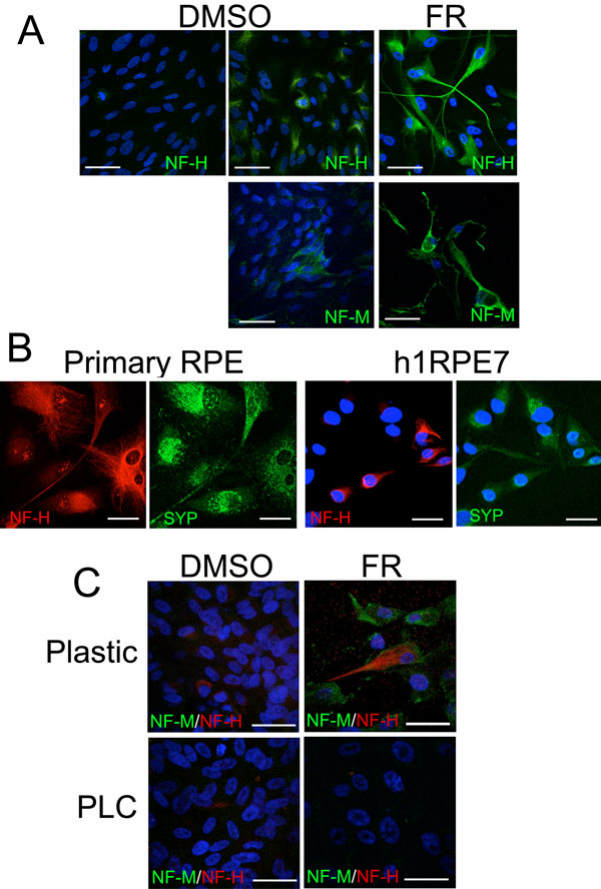
Neuronal markers are expressed in retinal pigment epithelium cells in culture but are lost after culture on a porcine lens capsule membrane (PLC). The expression of neurofilament heavy and medium polypeptides (NF-H and NF-M respectively) varied in dimethyl sulfoxide (DMSO)-treated cells. **A**: The first two panels show the variation of NF-H expression within the same dish of DMSO-treated ARPE-19 cells, which is increased across the whole dish after fenretinide (FR) treatment. Similarly, the expression of NF-M varies within cells of the same field and is increased by fenretinide. **B**: Passage 2 primary human RPE cells and an additional human RPE cell line (h1RPE7) express the neuronal markers NF-H and synaptophysin (SYP). **C**: Culturing ARPE-19 on a PLC decreases the expression of neuronal markers in control cells and prevents the fenretinide-induced increase of neuronal markers. Protein staining is indicated by the color of the text (red or green). All scale bars equal 50 μm.

NF-H expression was observed in control-DMSO-treated cells; however its expression varied. In the same dish we observed patches of cells with low and high levels of NF-H expression (Figure 5A). Fenretinide treatment increased levels of expression across the dish with strong staining along the cellular projections. Similarly, NF-M expression was also observed in a small percentage of control cells with increased levels of expression observed after 7 days treatment with fenretinide. To ascertain whether the expression of neuronal cell markers is a phenomenon specific to ARPE-19 cells in culture, we examined neuronal protein expression in cultured passage 2 human RPE cells and in the immortalized human RPE cell line h1RPE7 (Figure 5B). We observed strong cytosolic expression of NF-H in primary RPE and in approximately 50% of h1RPE7 cells. Similarly, synaptophysin expression was observed in both passage 2 RPE and h1RPE7 cells. The expression of neuronal markers NF-M/H was decreased in ARPE-19 cell by culture on PLC, a surface conducive to RPE cell differentiation [30] (Figure 5C). Culturing cells on PLC also prevented the fenretinide-induced increase of NF-M and NF-H in ARPE-19 cells.

**Figure 6:**
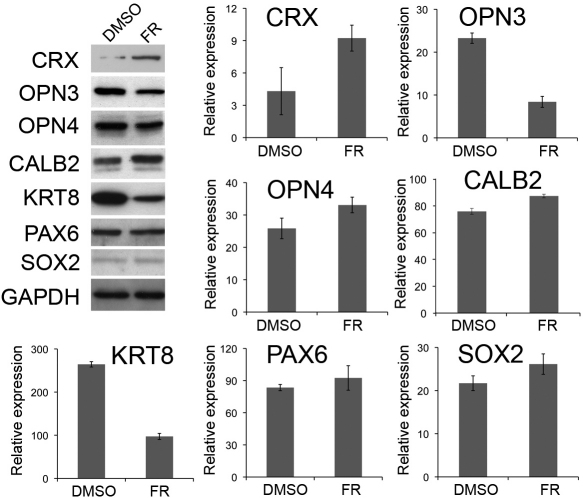
Detection of protein expression in ARPE-19 cells after dimethyl sulfoxide or fenretinide treatment by western blot analysis. Cells were treated with 3 μM fenretinide (FR) or dimethly sulfoxide (DMSO) for 7 days and extracts prepared for western blot. Equal amounts of protein were loaded onto a sodium dodecyl sulfate PAGE gel, transferred to membrane, and probed with antibodies generated against cone rod homeobox (CRX), opsin 3 (OPN3), melanopsin (OPN4), calbindin 2 (CALB2), cytokeratin 8 (KRT8), paired box 6 (PAX6), and sex determining region Y-box 2 (SOX2). Proteins were detected using chemiluminescence and visualized using autoradiographic film. Membranes were stripped and reprobed with glyceraldehyde 3-phosphate dehydrogenase (GAPDH) as a loading control. Representative blots from DMSO- and FR- treated cells are shown. Protein levels were quantified using densitometry and normalized relative to the GAPDH loading control. Data shown are mean±standard error of the mean normalized to GAPDH (n=4).

**Figure 7:**
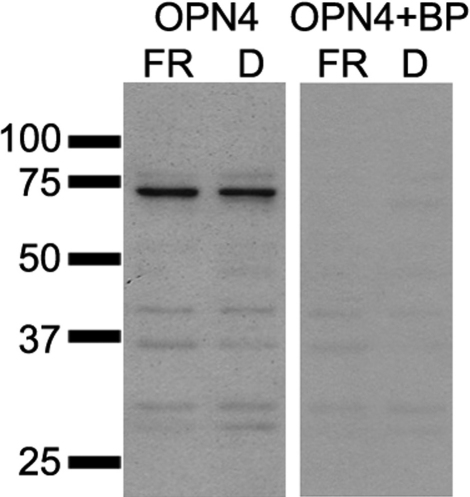
A melanopsin (OPN4) peptide-blocking experiment was performed to test the specificity of the antibody for western blot. Due to the presence of multiple bands on the OPN4 western blot the OPN4 antibody was pre-incubated with a fivefold excess of 15 amino acid N-terminal blocking peptide in 10% milk/TBS-T for 2 h at room temperature before hybridization. Proteins extracted from fenretinide (FR) and dimethyl sulfoxide (DMSO) treated cells were incubated with OPN4 antibody pre-incubated with the blocking peptide (OPN4+BP) or OPN4 antibody alone. A single band of approximately 70 kDa specific to OPN4 was absent in the western blot containing the blocking peptide.

Protein expression changes induced by fenretinide were examined in whole cell extracts by western blot analysis (Figure 6). A single band of the correct molecular weight was detected for each protein except for OPN4, where multiple bands were detected. In this instance we used a blocking peptide to identify the specific band (Figure 7). Individual bands were analyzed by densitometry and quantified relative to a GAPDH loading control. Fenretinide induced a significant increase in the expression of CRX, OPN4, and CALB2 proteins (all p<0.05, Student *t* test) and a decrease in the expression of OPN3 and KRT8 (p<0.001 and p<0.01, respectively, Student *t* test). We found no significant difference in the expression of PAX6 or SOX2 by western blot following fenretinide treatment (p=0.476 and 0.180, respectively).

## Discussion

Previous studies have shown that the human RPE cell line, ARPE-19, is capable of developing neuronal cell-like characteristics following treatment with fenretinide. The phenotypic changes were accompanied by an increase in non-retinal specific neuronal cell markers. Given that ARPE-19 express neuronal cell markers after treatment with fenretinide, we have investigated the potential for transdifferentiation toward a retinal phenotype in this cell line by examining the expression of retinal-associated markers after fenretinide treatment.

Following 7 days of treatment with 3 μM fenretinide, cells were morphologically similar to neurons, with an elongated cell body and the formation of neurite branching. Confirming previous findings [26,28], we found that neuronal cell markers were upregulated in fenretinide-treated cells. We also found that DMSO-treated control ARPE-19 cells expressed many of the neuronal cell markers examined, such as SYP, NSE, NF-M, NF-H, CALB2, and TUBB3. Mammalian adult RPE cells have been shown to switch on TUBB3 within 5 days of primary culture and a multitude of neuronal progenitor and neuronal markers within 3 passages in culture [33,34]. ARPE-19 cells are a highly proliferative cell line that have lost several key RPE cell characteristics, including pigmentation, the typical RPE cell cobblestone-like morphology, junction formation [24], and many key RPE proteins critical to normal cell function [35,36]. Loss of phenotypic characteristics, such as enzyme activity and polarization, is not limited to the ARPE-19 cell line, it has also been demonstrated in the human D407 RPE cell line [37]. Although previous studies have shown that ARPE-19 cells express RPE65 mRNA transcripts [24,25], RPE65 protein has yet to be observed [36]. The loss of RPE cell markers and phenotype accompanied by expression of neuronal cell markers in RPE cells suggests that cells de-differentiate away from the fully differentiated RPE cell phenotype in culture.

Subsequent to retinal injury in amphibians, RPE cells re-enter the cell cycle and proliferate into de-differentiated cells, losing pigmentation and many RPE-specific genes, while gaining neuronal and retinal progenitor cell markers [38,39]. ARPE-19 cells appear to have undergone this initial transformation under normal culture conditions. We found that many genes involved in the differentiation of the neural retina are expressed in ARPE-19 cells, including *Crx*, a homeobox transcription factor expressed in developing and mature photoreceptor cells, which is required for photoreceptor differentiation, [40] and *NRL*, a leucine zipper transcription factor involved in the development of, and expressed predominantly in, rods [41]. These two transcription factors bind with the orphan nuclear receptor NR2E3 to regulate photoreceptor cell differentiation [42]. The presence of these factors in ARPE-19 cells under normal culture conditions suggest that they are predisposed toward a photoreceptor cell phenotype. Previous studies have shown that *Crx* is upregulated in human retinoblastoma cells after treatment with retinoids [43,44], and here we show that a synthetic retinoic acid also increases CRX gene and protein expression in human RPE cells.

ARPE-19 cells express several key transcription factors implicated in the maintenance of retinal progenitor cell multipotency, including PAX6 [45] and SOX2 [46]. PAX6, a master control transcription factor essential for normal development of the eye, is sufficient to induce proliferation, depigmentation, and neuronal gene expression in chick RPE cells [47]. PAX6 has been identified in transdifferentiating nonpigmented RPE [48], developing RPE [49], cultured adult RPE, and in human embryonic stem cell-derived RPE cells in vitro [35] but not in vivo [35,48]. The multipotency of ARPE-19 is further implied by the expression of SOX2, a transcription factor associated with the proliferation of neural retina progenitor cells [46] and pluripotency [50,51]. Our findings, showing that PAX6 and SOX2 are present in ARPE-19 cells, suggest that these cells retain plasticity in terms of their capacity to differentiate.

While the expression of neuronal cell markers has previously been reported in ARPE-19 [28], these are the first data to demonstrate the presence of retinal cell-associated markers in a mammalian RPE cell line. Many of the retinal cell markers we examined are detectable under control conditions in ARPE-19 cells, including markers for photoreceptors, and neuroretinal cells, including retinal ganglion cells, amacrine cells, and retinal astrocytes. Importantly, fenretinide was able to induce the expression of the red cone opsin Opn1lw in ARPE-19 cells, which is the first report of such an occurrence in a mammalian RPE cell line; however we were unable to detect rhodopsin expression in either group. It is possible that fenretinide treatment does not induce the expression of rhodopsin in these cells, similar to findings after retinoic treatment in retinoblastoma cells by Li et al. [44]. We suggest that the retinoid treatment performed in the current study may be conducive to cone differentiation, while inhibiting rod gene expression. Alternatively, the lack of detectable rhodopsin may reflect a conserved developmental timing system where the differentiation of retinal cells in culture reflects that observed in vivo during development [52].

The RPE expresses many visual pigment-like proteins despite its apparent lack of light responsiveness. Our finding that melanopsin (OPN4) is expressed in ARPE-19 cells together with Opn1mw/lw is of significant interest as unlike the latter, melanopsin is a novel photopigment that shares a common ancestry with invertebrate rhabdomeric proteins [53]. Unlike the classic visual opsins, melanopsin is not thought to be expressed in rod and cone cells in the retina, instead its expression is restricted to networks of intrinsically photoreceptive retinal ganglion cells (ipRGCs) where it functions in nonimage-forming light responses [54-56]. Expression of melanopsin mRNA has previously been reported in the RPE cells of *Xenopus* [57], mouse [58], and humans; however for the latter it was suggested that this could be attributed to retinal contamination during dissection of tissues [59]. This is the first reporting of melanopsin expression in a human cell line; however immunoreactivity is confined to the nucleus, whereas it is localized throughout the cell membrane of ipRGCs in the rodent, primate, and human retina [56,60] and after overexpression in the human RPE cell line D407 [61]. Although melanopsin expression is increased by fenretinide in our cells staining for the RGC marker, BRN3 was not detected (data not shown). Previous findings have suggested that BRN3 is not required for the initial specification of RGCs, but it is needed for maturation of RGC cells [62]. The absence of BRN3 staining may reflect the transdifferentiation of cells toward an immature RGC, which might also explain the discrepancy in the localization of melanopsin. Alternatively, nuclear expression of melanopsin in the ARPE-19 cells line could indicate that the cells do not produce a mature form of the protein, which localizes to the plasma membrane [61]. Given these findings, the ARPE-19 cell line could prove to be a useful model for the study of melanopsin trafficking in human cells.

Other non-canonical opsins are also expressed outside of the photoreceptor cell layer of the retina. Human encephalopsin (Opn3) is a novel opsin of unknown function expressed in a wide range of non-neuronal tissues and the brain and retina [63]. In the eye it is present from early developmental stages to adulthood [64]. Here we show that encephalopsin is expressed in ARPE-19 cells and that both mRNA and protein levels are decreased after fenretinide treatment, which suggests that differentiation away from the epithelial cell type is not conducive to its expression. As yet Opn3 has not been identified in RPE cells in vivo.

Two of the non-canonical opsins, RRH and RGR opsin, are expressed in RPE cells in vivo. RRH retinal pigment epithelium-derived rhodopsin homolog (peropsin) is localized within the microvilli of RPE cells [65] where it is thought to act as a photoisomerase [66]. A previous study has shown that retinoic acid can delay the expression of RRH in ARPE-19 cells for up to 5 weeks [25]; however expression of the *Rrh* gene in our cells was unaffected by fenretinide treatment, which again suggests that either the synthetic retinoid may act on a different signaling pathway or that the passage number of ARPE-19 cells is a key factor in the regulation of some RPE-specific genes in culture. RGR opsin is a G protein-coupled receptor found in RPE and Muller cells [67] and acts as a photoisomerase, converting all-trans retinal to 11-cis retinal [68]. We were unable to detect *Rgr* mRNA expression in ARPE-19 cells. This absence, which has also been described previously [69], provides further evidence to suggest that ARPE-19 cells are in a de-differentiated state.

Recently, Samuel et al. identified a signaling pathway responsible for fenretinide-induced neuronal differentiation of ARPE-19 cells [70]. Fenretinide treatment was shown to activate the signaling kinases c-Raf and MEK1/2 in cells, while blocking MEK1/2 with inhibitors and small interference RNA (siRNA) prevented neuronal differentiation. The MEK pathway regulates many cellular processes, including proliferation and differentiation. Importantly, this pathway has also been implicated in the transdifferentiation of RPE to retina in vivo in adult newt *Xenopus* larvae and chick embryo [14,71,72].

The possibility of using transdifferentiated human RPE cells for the treatment of retinal disease is somewhat controversial. Clinical treatments for retinal degenerative diseases, resulting in the loss of photoreceptor cells, are extremely limited at present. Recently, there has been an increased interest in the development of cell-based therapies, which aim to either halt the progression of photoreceptor cell death or replace cells lost as a consequence of disease. To induce the transdifferentiation of a patients’ own RPE cells to form a fully functional retina, as observed in other species, would be an ideal solution to treat many forms of retinal degeneration. Although the transdifferentiation of mammalian RPE cells into retinal cells does not naturally occur in vivo, it might be possible to manipulate this process after retinal injury. It would therefore be interesting to view the effects of fenretinide treatment in the eye after retinal injury or in a degenerating animal model. The PLC will also provide a useful in vitro model system to study the effects of the microenvironment on fenretinide-induced transdifferentiation of RPE cells. Although not the natural substrate of RPE, the PLC could be used to provide clues as to the identity of signaling molecules required for, or the mechanisms preventing transdifferentiation of mammalian RPE in vitro and RPE cell differentiation in vivo in response to injury.

In this study we have demonstrated that in vitro ARPE-19 cells have the potential to differentiate toward a retinal cell lineage. At this stage it is unclear whether complete transdifferentiation, as specified by the formation of a fully functional cell, is a possibility. It is unlikely that the signals required for full transdifferentiation of cells are present in a tissue culture dish, and ultimately the local microenvironment of the damaged retina would be expected to play a key role in directing cell fate. A comparative analysis of the RPE response to retinal injury between species is imperative to identify the transdifferentiation signaling pathways absent in most vertebrates.
